# Clinical Updates in Pulmonary Hypertension

**DOI:** 10.31083/RCM48374

**Published:** 2026-06-08

**Authors:** Gabriela Diaz, Alyssa Lawrence, Julia Ossi, Dirichi Arungwa, Allison Weiss, Hafeez Ul Hassan Virk, Muzamil Khawaja, Chayakrit Krittanawong

**Affiliations:** ^1^Department of Internal Medicine, Emory University, Atlanta, GA 30322, USA; ^2^Division of Cardiovascular Disease, Department of Medicine, Albert Einstein Healthcare Network, Philadelphia, PA 19141, USA; ^3^Department of Cardiology, Emory University, Atlanta, GA 30322, USA; ^4^HumanX, Delaware, DE 19801, USA

**Keywords:** pulmonary hypertension, PAH, PH

## Abstract

Pulmonary hypertension is a heterogeneous, progressive condition affecting pulmonary vasculature. Epidemiologic studies have highlighted underdiagnosis and persistent disparities in access to expert care, treatments, and outcomes. Biomarkers and imaging‑based phenotyping are enhancing early detection, while emerging technologies for monitoring and novel treatment targets show promise for a new horizon in individualized management of pulmonary hypertension. This review aims to summarize the epidemiology, pathogenesis, definitions, treatment, and future directions of pulmonary hypertension.

## 1. Introduction

Pulmonary hypertension (PH) is a complex and progressive disorder characterized by elevated blood pressure within the pulmonary arteries, leading to increased right ventricular (RV) afterload and right heart failure [1, 2]. PH encompasses a heterogeneous group of conditions classified into 5 distinct groups based on etiology and pathophysiological mechanisms, as defined by the World Health Organization (WHO) [1, 2]: pulmonary arterial hypertension (PAH), PH due to left heart disease (LHD), PH arising from lung diseases, chronic thromboembolic PH (CTEPH), and PH related to unclear or multifactorial causes. The prevalence of PH globally is estimated to be 1% [3, 4] and the disease is associated with substantial socioeconomic burden, emphasizing the importance of enhanced recognition and clear management pathways [5].

Advances in imaging techniques, biomarkers, and functional testing can assist in the identification of PH and differentiation among its subtypes to guide treatment and improve outcomes [2, 6]. A multidisciplinary approach involving input from cardiologists, cardiothoracic surgeons, pulmonologists, and palliative care can aid in comprehensive care for PH patients. This review aims to provide an overview of the pathophysiological mechanisms, diagnostic tools, and treatment modalities of PH to optimize morbidity and mortality and cover emerging and future research into novel therapeutic interventions.

## 2. Definitions

Currently, PH is defined as a mean pulmonary arterial pressure (mPAP) greater than 20 mmHg at rest and a pulmonary vascular resistance (PVR) greater than 2.0 Wood units (WU) on right heart catheterization (RHC) [1, 2]. These thresholds are relatively new. In 2018, a task force at the 6th World Symposium on Pulmonary Hypertension (WSPH) suggested lowering the mean pulmonary arterial pressure (mPAP) threshold to 20 mmHg and introducing a PVR cutoff of greater than 3 WU [7]. Originally, PH was defined by a mPAP ≥25 mmHg and did not consistently include PVR as a parameter [8]. This definition was first published in 1973 by the WHO and was notably decided upon arbitrarily. Challenges to this definition were informed by developments in clinical research focused on the mortality risk associated with mPAP at varying levels. Landmark studies not only established normal mPAP levels but also explored the increase in mortality associated with an mPAP >20 mmHg and found that elevation of mPAP in the range between 21 and 24 mmHg is an independent predictor of mortality. Thus, lowering the hemodynamic thresholds was introduced for diagnosing PH earlier in the disease course [8, 9].

While this may improve mortality and decrease disease burden, consideration should be given to how these definition changes impact resource utilization and could lead to overdiagnosis. In clinical practice, shifting the hemodynamic criteria has led to increased use of RHC and increased referrals to specialized PH centers [10]. Although this does not inherently translate to overdiagnosis or improper resource allocation, it has called into question if the same approaches used in moderate and severe PH should be applied to individuals with mild PH (mPAP 21 to 24 mmHg). There are gaps in inclusion of this population in recent landmark clinical trials, limiting the application of these findings to mild PH.

PH is categorized by the location of vascular and/or organ involvement, starting from the pulmonary arteries, followed by the pulmonary capillary bed surrounding the lung alveoli, and into the left side of the heart. Pulmonary capillary wedge pressure (PCWP) and PVR distinguish these classifications from one another. PCWP, provides an estimate for left ventricular end-diastolic pressure and thus an indirect estimate for left ventricular (LV) preload and left atrial (LA) pressure. Normal range for PCWP is 4–12 mmHg [10].

Precapillary PH refers to PH caused by remodeling of the pulmonary vasculature in the absence of LHD and is defined by a PCWP ≤15 mmHg and PVR >2 WU. Conversely, postcapillary PH refers to increased pulmonary venous pressure caused by LHD and is defined by a PCWP >15 mmHg and a PVR ≤2 WU. Combined precapillary/postcapillary PH is defined by both a PVR >2 WU and a PCWP >15 mmHg and includes PH caused by both LHD and lung disease [1, 2, 10]. See Table 1 (Ref. [2, 7, 11]) for a summary of PH hemodynamic definitions.

**Table 1.** **Hemodynamic definitions of PH by organization**.

Organization	Hemodynamic definitions based on RHC		Clinical group involvement	Key differences
WSPH (2018) [7]	Precapillary PH	mPAP >20 mmHg	1, 3, 4, and 5	In 2018, the WSPH Symposium proposed the change from mPAP ≥25 mmHg to >20 mmHg.
		PAWP ≤15 mmHg		
		PVR ≥3 WU		
	Isolated Postcapillary PH	mPAP >20 mmHg	2 and 5	WSPH believes more information and research are required in the area of exercise PH and does not provide a hemodynamic definition.
		PAWP >15 mmHg		
		PVR <3 WU		
	Combined Precapillary and postcapillary PH	mPAP >20 mmHg	2 and 5	
		PAWP >15 mmHg		
		PVR ≥3 WU		
AHA/ACC (2023) [11]	PH	mPAP >20 mmHg	All	Provides guidance on perioperative management and risk stratification of PH.
	Precapillary PH	mPAP >20 mmHg	1, 3, and 4	
		PAWP ≤15 mmHg		
		PVR >2 WU		
	Isolated Postcapillary PH	mPAP >20 mmHg	2	
		PAWP >15 mmHg		
		PVR ≤2 WU		
	Combined precapillary and postcapillary PH	mPAP >20 mmHg	2 and overlap between 2 and 3	
		PAWP >15 mmHg		
		PVR >2 WU		
	Exercise PH	mPAP/CO slope between rest and exercise >3 mmHg/L/min	Exertional dyspnea with preserved LV ejection fraction with normal resting PAWP	
ESC/ERS (2022) [2]	PH	mPAP >20 mmHg	None delineated	Presents a diagnostic algorithm with echocardiography or CPET as an initial cardiac study in suspected PH.
	Precapillary PH	mPAP >20 mmHg		
		PAWP ≤15 mmHg		
		PVR >2 WU		
	Isolated Postcapillary PH	mPAP >20 mmHg		
		PAWP >15 mmHg		
		PVR ≤2 WU		
	Combined precapillary and postcapillary PH	mPAP >20 mmHg		
		PAWP >15 mmHg		
		PVR >2 WU		
	Exercise PH	mPAP/CO slope between rest and exercise >3 mmHg/L/min		

RHC, right heart catheterization; CO, cardiac output; mPAP, mean pulmonary arterial pressure; PAWP, pulmonary arterial wedge pressure; PH, pulmonary hypertension; PVR, pulmonary vascular resistance; WU, Wood units; CPET, cardiopulmonary exercise testing; LV, left ventricular; WSPH, World Symposium on Pulmonary Hypertension; AHA/ACC, American Heart Association/American College of Cardiology; ESC/ERS, European Society of Cardiology/European Respiratory Society.

Once a diagnosis of PH is established by hemodynamic thresholds, it is further classified into one of five WHO groups, each of which has distinct mechanisms and clinical implications, making accurate classification essential for diagnosis and management.

Group 1, PAH, is a vasculopathic condition that can be heritable, drug-induced, or related to conditions such as connective tissue diseases, human immunodeficiency virus (HIV), and congenital heart disease. It comprises around 2% of PH and disproportionately affects women [3, 12]. Of note, PAH carries a mortality rate of nearly 50% over 5 years [2].

Group 2 PH is the most common form encompassing around 70% of PH and results from LHD including heart failure with either reduced or preserved ejection fraction and valvular diseases [13].

Group 3 PH is implicated in 10% of PH and arises from lung diseases and/or hypoxia, such as chronic obstructive pulmonary disease (COPD), interstitial lung diseases (ILD), sleep-disordered breathing, and chronic high-altitude exposure. This form of PH is generally mild to moderate but can be severe, particularly in ILD [14].

Group 4, CTEPH, comprises 1–3% of PH and is caused by unresolved pulmonary emboli that lead to vascular obstruction and remodeling [15].

Group 5 PH encompasses approximately 15% of PH and includes cases with unclear or multifactorial causes, such as those related to hematologic disorders (e.g., sickle cell disease), chronic myeloproliferative diseases, post-splenectomy, sarcoidosis, metabolic disorders including thyroid disease, and chronic kidney disease [16].

## 3. Pathophysiology

An elevated mPAP reflects downstream vascular, cardiac, and systemic pathology and the same hemodynamic profile can arise from very different disease pathways. Understanding the pathophysiology of PH allows for the initiation of targeted therapies.

At the cellular level, PH is caused by the remodeling of the pulmonary vasculature. This remodeling causes increased PVR and eventually leads to RV hypertrophy and dysfunction. PAH is marked by the remodeling of the precapillary arterioles. In contrast, remodeling of postcapillary vessels is the hallmark of conditions such as scleroderma-associated PAH, pulmonary veno-occlusive disease, pulmonary capillary hemangiomatosis, and Group 2 PH [5, 17].

Vascular remodeling happens in all three layers: the intima, media, and adventitia. Several different factors, including genetic predisposition, hypoxia, and high shear stress trigger these processes. These factors lead to dysregulation of the endothelial cells that line the vasculature, smooth muscle cells, and fibroblasts. Unregulated growth and loss of endothelial cell integrity cause platelet activation and thrombosis of these small arteries [18]. In CTEPH, this remodeling may be responsible for PH that persists even after pulmonary endarterectomy [19].

Genetic mutations play a role in the pathophysiology of certain groups of PH. Transforming growth factor-β (TGF-β) is a family of signaling proteins that play a major role in PAH. Bone morphogenetic protein receptors (BMPR) are members of this signaling protein family. Heterozygous mutations in BMPR2 specifically are seen in 80% of familial PAH and 20% of idiopathic PAH [20, 21].

Targeting mutations to develop PAH therapies is a promising and emerging field of research [20, 22].

**Fig. 1.**
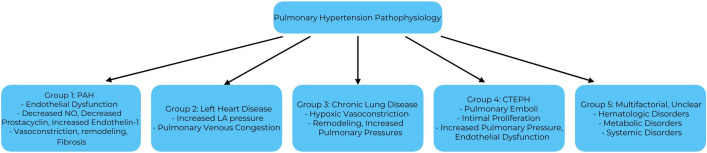
**Pathophysiology summary of PH groups**. PAH, pulmonary arterial hypertension; NO, nitric oxide; LA, left atrial; CTEPH, chronic thromboembolic pulmonary hypertension.

See Fig. 1 for a summary of WHO PH Groups and their pathophysiology.

## 4. Clinical Presentation

Most symptoms of PH are nonspecific and related to RV dysfunction. Symptoms include dyspnea, angina, syncope, and generalized fatigue, and mainly occur with exertion. These non-specific symptoms can lead to a delay in diagnosis. As little as a two-year delay in PH diagnosis is linked to substantial and potentially not intervenable disease burden [1]. As the disease progresses, right heart failure advances and causes symptoms such as lower extremity edema and abdominal distension [23]. Less common symptoms include hemoptysis and hoarseness. Hemoptysis is mediated by abnormal blood flow in the pulmonary vascular system, which causes rupture of the bronchial arteries. Hoarseness is caused by compression of the recurrent laryngeal nerve by a dilated pulmonary artery (PA). This dilation can also cause wheezing, coughing, and in severe cases, the PA may rupture, causing cardiac tamponade [23].

Physical findings include an increased pulmonic component of the second heart sound, an S3, tricuspid regurgitation murmur, and pulmonary regurgitation murmur. As discussed above, as disease severity progresses and pulmonary pressures increase, exam findings represent RV overload such as jugular venous distention, edema, ascites, and hepatomegaly. If left untreated, the progression to RV failure leads to death. This is due to increased PVR causing RV hypertrophy, which can be either maladaptive (eccentric) or adaptive (concentric) [17]. Furthermore, studies have shown that RV contractility differs between subcategories, with idiopathic and congenital PAH experiencing hypercontractility, while scleroderma-associated PAH experiences hypocontractility [24]. Mechanisms that drive these changes in the RV are not fully understood; thus, there is a current lack of therapies that target RV remodeling specifically.

Though initial symptoms may be vague, clinicians should consider PH as a differential when seeing patients at risk. Such patients include those with substance use disorder, prior PE, limited cutaneous systemic sclerosis, HIV, and primary liver disease [1].

## 5. Diagnosis 

**Fig. 2.**
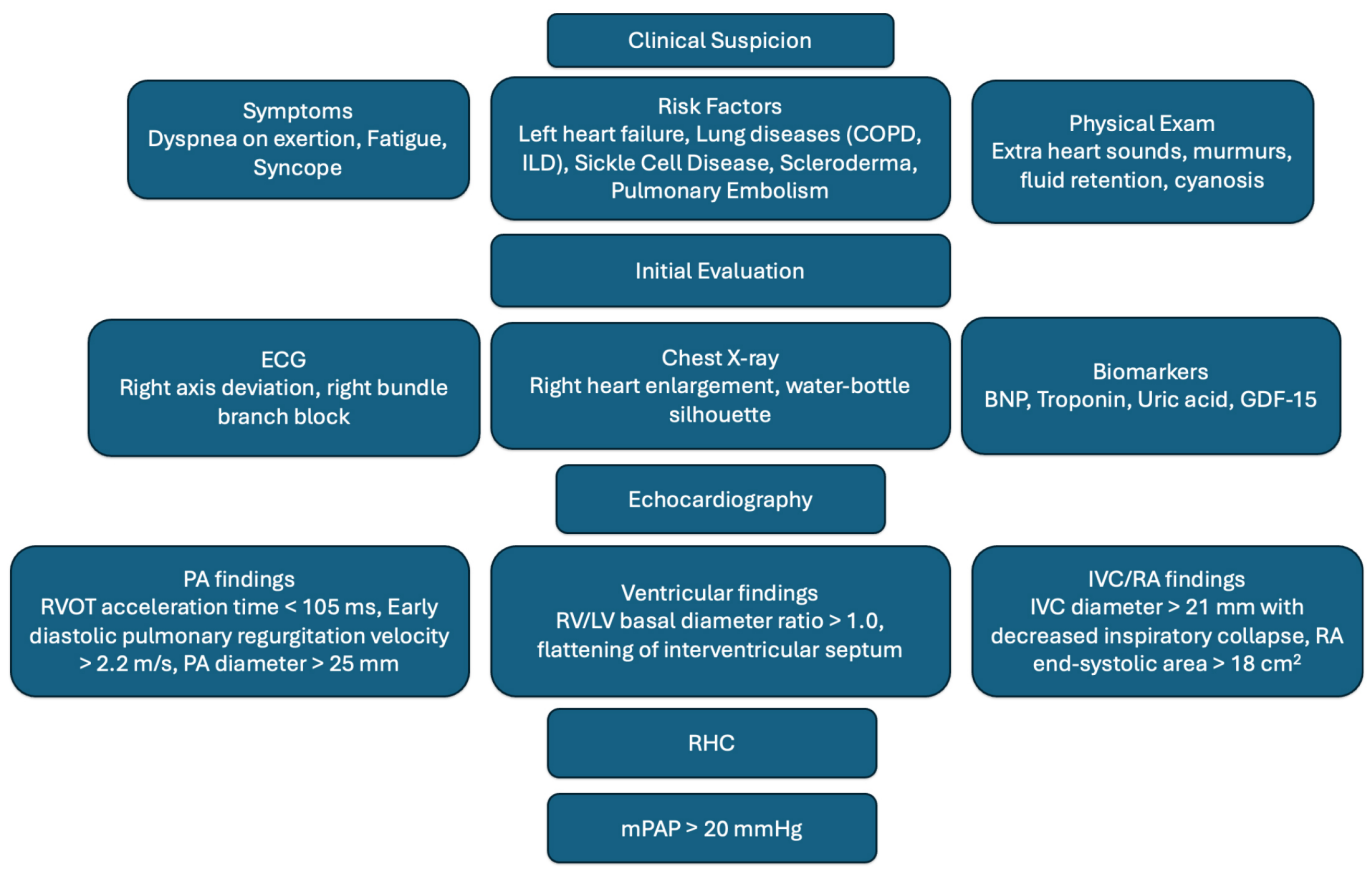
**Diagnostic algorithm for PH (clinical → imaging → RHC)**. COPD, chronic obstructive pulmonary disease; ILD, interstitial lung disease; ECG, electrocardiogram; BNP, brain natriuretic peptide; GDF, growth differentiation factor; PA, pulmonary artery; RVOT, right ventricular outflow tract; RV, right ventricle; LV, left ventricle; IVC, inferior vena cava; RA, right atrium; RHC, right heart catheterization; mPAP, mean pulmonary arterial pressure.

Diagnosing PH requires a comprehensive set of investigations initiated after suspicion based on symptoms and physical examination. Several diagnostic algorithms have been proposed, such as the European Respiratory Society (ERS) PH diagnostic algorithm [2], which recommends screening patients with signs and symptoms of PH with a transthoracic echocardiogram (TTE) and stratifying findings into low, intermediate, and high probability of PH. In patients with an intermediate or high probability, risk factors for PAH, or a history of pulmonary embolism, ERS recommends an expedited referral to a PH expert center for multimodality testing including RHC. See Fig. 2 for a diagnostic algorithm. This approach is more common in Europe compared to the United States due to a combination of socioeconomic factors including structural barriers (insurance, geography, transportation) which limit access to expert PH centers, genetic testing, and advanced imaging modalities [11].

## 6. Imaging

### 6.1 Transthoracic Echocardiogram (TTE)

In many patients, TTE is utilized to evaluate dyspnea, and is therefore a useful screening tool in the primary or ambulatory care setting. PH should be considered in patients with TTE findings of a tricuspid regurgitant jet velocity >2.8 m/s, corresponding to a PA systolic pressure of approximately 35 mmHg [1]. Though elevated PA systolic pressure via TTE does not predict the PH group and should not be used alone to assess for PH, high PA pressures in the setting of a small LA volume can suggest Group 1 PH, and findings of LHD on TTE can suggest Group 2 PH [23].

Advanced TTE findings may also be used to determine the likelihood of PH. Although these findings may suggest PH, clinical judgment is required to determine which patients should proceed to RHC for definitive diagnosis and which patients may have these findings due to other causes. When evaluating the ventricle, an RV/LV basal diameter ratio >1.0 and flattening of interventricular septum suggest elevated right-sided pressures. PA-specific findings include RV outflow Doppler acceleration time <105 ms and/or midsystolic notching, early diastolic pulmonary regurgitation velocity >2.2 m/s, and PA diameter >25 mm. Finally, inferior vena cava (IVC) and RA findings include IVC diameter >21 mm with decreased inspiratory collapse and RA end-systolic area of >18 cm^2^. To increase the probability of PH, findings from at least two of the three categories should be present [2, 25, 26]. As PH worsens, there can be signs of RV dysfunction on TTE as determined via decreased tricuspid annular plane systolic excursion or RV fractional area change [23]. Echocardiography, although useful as a screening tool, is dependent on many factors to obtain reliable measurements including patient body habitus, technician skill, and inter-reader variability. If there is a high clinical suspicion of PH, further workup should be pursued.

### 6.2 Cardiac MRI

Use of cardiac MRI in PH is promising as it provides information on hemodynamics of the PA and RV. Studies have found utility in calculating the RV ejection fraction, stroke volume index, and RV end systolic volume index from cardiac MRI to stratify PH patients into various risk categories [2, 27]. These values can be incorporated into a PAH risk assessment tool such as the ERS/ESC Baseline Risk Score to prognosticate and determine aggressiveness of treatment [2]. Small studies have shown applicability of these risk assessment tools in other PH groups as well [2, 28]. In PH, RV enlargement with stroke volume reduction as seen on cardiac MRI can precede the clinical manifestation of right heart failure and promote earlier interventions. Cardiac MRI can also be used to noninvasively monitor RV function and treatment response over time. Notably, cardiac MRI remains costly with limited regional availability and clinician comfort with the modality but may be a useful tool in the diagnosis and monitoring of PH with further validation.

### 6.3 Right Heart Catheterization

Though diagnosis may be suggested based on the above measures, RHC is the gold standard for diagnosis and classification via direct measurement of mPAP, PCWP, and PVR [1]. Of the hemodynamic parameters, PCWP is a crucial value that must be carefully obtained and interpreted, as some patients with normal systolic function and poor diastolic function, may have a high normal or only slightly elevated PCWP. Additionally, over- or under-wedging, failure to measure at end-expiration, or improper leveling of the transducer can lead to inaccurate PCWP measurements [29, 30]. Exercise testing and cardiac output calculation via thermodilution or Fick’s equation can help confirm RHC findings and classification.

In patients whose RHC suggests pre-capillary PH, acute vasoreactivity testing should then be performed to identify if vasodilators may be helpful in treatment. If the mPAP decreases meaningfully without decreasing the cardiac output, then the patient is considered a positive acute responder [31]. These patients are the most likely to experience a sustained response to high doses of calcium-channel blockers for treatment of PAH [32]. Care must be taken during acute vasoreactivity testing, as it may precipitate pulmonary edema in patients with Group 2 PH or right heart failure [30].

Performing RHC for diagnosis should be done at expert PH centers to minimize the risks and need for multiple procedures. The most common risks of RHC are hematoma at the puncture site, venous access complications such as pneumothorax, induction of supraventricular tachycardia (SVT), or hypotension from vagal reactions or vasoreactivity testing [29]. Given the need for accurate values, multiple measurements, performance at expert centers, and careful technique are required to minimize error and improve validity of the RHC [29]. The need for invasive testing to diagnose PH poses difficulties as patients in rural or otherwise under-resourced settings without access to a catheterization lab or advanced testing may lack the resources needed to achieve an accurate diagnosis.

## 7. Biomarkers

Though biomarkers alone are not sufficient to make a diagnosis of PH, they may be used to identify those at risk of PH prior to RHC and then prognosticate after the diagnosis is made. Many biomarkers can represent changes in cardiac function, but the strongest present predictor of prognosis in PH is *N-*terminal prohormone of brain natriuretic peptide (NT-proBNP). NT-proBNP and BNP correlate with myocardial dysfunction and are therefore widely used to monitor disease progression [1, 2, 23, 32]. Other biomarkers include high-sensitivity troponin, uric acid, as well as emerging biomarkers such as growth factor differentiation-15 (GDF-15). In some studies, high-sensitivity troponin has been present in 95% of patients with PAH, with a direct stepwise increase in mortality as troponin increases. In fact, patients with PAH without troponin elevation had the lowest risk of death compared to all other groups. These findings suggest PH is associated with injury in cardiac myocytes that progresses as the disease worsens [33]. Regarding uric acid, experimental models have shown that higher uric acid levels are associated with worse PH hemodynamic measurements, and it may have a negative relationship with survival [33, 34]. The proposed mechanism is that uric acid promotes smooth muscle growth in experimental models [33, 34, 35]. Given these findings, uric acid level may be a novel therapeutic target in patients with PAH. The final emerging biomarker is GDF-15, which has been associated with increased risk of mortality, lower 6-minute walk distance (6MWD), higher right atrial pressure (RAP), and higher NT-proBNP. A prospective cohort study showed elevated levels of GDF-15 were significantly and independently associated with an increased risk of mortality or lung transplantation in idiopathic PAH [36]. Ultimately, once a diagnosis is confirmed, PH is divided into four functional classes by the WHO (WHO-FC) which were modeled on the New York Heart Association (NYHA) heart failure functional classes, with a higher class equating to more severe disease. The WHO-FC classes provide a useful insight on how PH symptoms impact a patient’s quality of life. Change in WHO-FC is commonly used as a metric in clinical trials to determine efficacy [37]. Notably, the CHEST guidelines focus heavily on the WHO-FC to stratify treatment recommendations [38]. WHO-FC Class I describes patients with PH but without resulting limitation of physical activity. Class II patients experience a slight limitation of physical activity. They are comfortable at rest. Class III patients experience marked limitation of physical activity. They are comfortable at rest. Less than ordinary activity causes undue dyspnea or fatigue, chest pain, or near syncope. Class IV patients are unable to carry out any physical activity without symptoms and dyspnea and/or fatigue may be present at rest. Treating PH is complex and it is imperative to consider values, goals, cost, and quality of life when suggesting therapies.

## 8. Management

Over the past two decades, the management of PH—particularly PAH—has been transformed by a series of landmark clinical trials that established mechanism-targeted therapy as the foundation of care. PH therapies aim to lower pulmonary pressures, modify vascular dysfunction, preserve right ventricular function, improve quality of life, and extend survival [1, 8]. We will present management stratified by the WHO PH group but recognize that tailoring treatments to both mechanisms and individual patient circumstances is the way forward.

### 8.1 Group 1: PAH

There are five main classes of medications that have been approved for the treatment of PAH: endothelin receptor antagonists (ERAs), soluble guanylate cyclase stimulators, phosphodiesterase-5 inhibitors (PDE5i), prostacyclin analogs/agonists, and the pivotal activin signaling inhibitors. The first, ERAs, inhibit endothelin-1, a potent vasoconstrictor, which decreases pulmonary vascular resistance. The first oral dual ERA approved for PAH was bosentan, which demonstrated improvements in exercise capacity, hemodynamic measurements, and time to clinical worsening in the ‘BREATHE-1’ and ‘study 351’ trials [39, 40]. Shortly after, the ARIES trial found improvements in exercise capacity with the ERA ambrisentan [41]. The landmark SERAPHIN trial of macitentan, a dual ERA with enhanced tissue penetration, was the first large, long-term, event-driven study to establish that ERA therapy significantly reduces the composite risk of morbidity and mortality compared with placebo in PAH, providing evidence for both monotherapy and combination use in Group 1 disease, since the majority of patients were on a PDE5i prior to enrollment [42]. Practical differences between bosentan, ambrisentan, and macitentan include once versus twice daily dosing and degree of LFT derangement [43].

PDE5i agents such as sildenafil and tadalafil promote vasodilation by inhibiting the breakdown of cyclic guanosine monophosphate (cGMP). They have been proven to improve exercise tolerance and delay clinical disease progression in several clinical trials [44, 45]. The AMBITION trial showed lower rates of death, hospitalization for worsening PAH, disease progression, or unsatisfactory long-term clinical response with initial combination therapy with ambrisentan and tadalafil when compared to ambrisentan or tadalafil monotherapy. Notably, sildenafil is frequently cited as the most cost-effective first-line oral therapy for PAH [46].

Prostacyclin analogs (epoprostenol, treprostinil, and iloprost) and agonists (selexipag) promote vasodilation, inhibit platelet aggregation and slow/reverse vascular remodeling. They also confer a survival benefit, particularly in advanced PAH [47]. The GRIPHON trial demonstrated a delay in clinical worsening when selexipag was added to monotherapy while the TRIUMPH and AIR trials demonstrated significant changes in 6MWD at week 12 with the use of inhaled treprostinil and iloprost compared to placebo [47, 48, 49].

Soluble Guanylate Cyclase Stimulators such as riociguat work by increasing cGMP levels. They have been shown to improve exercise tolerance and pulmonary vascular resistance in PAH, especially related to connective tissue disease [50].

Sotatercept was the first activin inhibitor approved by the Food and Drug Administration (FDA) for the treatment of PAH, and it inhibits activin, which is a part of the TGF-β superfamily and is involved in proliferation of endothelium and smooth muscle cells, leading to PA remodeling. This drug was a significant accomplishment for PAH, where existing therapies focused on symptom control rather than addressing the underlying cause of vascular remodeling. The PULSAR, ZENITH, and STELLAR trials demonstrated an improvement in parameters such as PVR, 6MWD, time to clinical worsening event (death, lung transplantation, or PAH-related hospitalization greater than 1 day), and WHO functional class, even in high-risk PAH [51, 52, 53]. Per the HYPERION trial, sotatercept decreased the risk of disease progression when added to background therapy within the first year of diagnosis [54]. The ongoing SOTERIA open-label extension study aims to evaluate the safety and tolerability of sotatercept when added to background PAH therapy, but interim results support similar rates of adverse effects as in the parent studies and improvements made during the parent trials were maintained at one year [55]. These advancements emphasize the value of continued research efforts in PAH and the hope for additional well-tolerated disease-modifying agents.

In PAH refractory to the above therapies, lung transplantation or atrial septostomy may be considered, though these are reserved for patients with advanced disease and poor prognosis and should be directed by expert PH clinician teams [2, 31].

The ESC guidelines recommend continuing high-dose calcium channel blockers in patients with idiopathic PAH, hereditary PAH, or drug- or toxin-associated PAH in WHO-FC I or II if they exhibit marked hemodynamic improvement [2]. In this population if there are no cardiopulmonary comorbidities and patients are treatment-naive, initial combination therapy with an ERA and PDE5i is recommended, and additional therapy with a prostacyclin analog as sequential combination therapy should be considered. The 2022 ESC guidelines also support the addition of an ERA to PDE5i or oral/inhaled prostacyclin [2].

### 8.2 Group 2: PH Due to LHD

LHD is the most common cause of PH and has limited options for management. Treatment of Group 2 PH focuses on the underlying etiology of LHD, which can include ischemic disease, heart failure with reduced or preserved ejection fractions, non-ischemic cardiomyopathy and valvular dysfunction. Diuretics play a role in symptom management for RV dysfunction causing fluid retention [2].

At the 2024 meeting of the 7th World Symposium on Pulmonary Hypertension, experts reinforced prior recommendations against the use of PAH medications for the treatment of PH-LHD, albeit as a class III recommendation given complexities in this population [56]. No large, well-powered randomized clinical trials have demonstrated the use of PAH medications in Group 2 PH. The EMBRACE-HF trial showed that SGLT2-i decreased PAP in patients across all spectra of heart failure independent of diuresis [57]. In a small study utilizing implanted PA pressure monitoring devices, switching from an angiotensin-converting enzyme inhibitor or angiotensin receptor blocker to sacubitril/valsartan resulted in an acute, significant decrease in PA pressures between 1 and 5 days post-initiation, though there was no information on follow-up nor other metrics to monitor clinical response [58].

### 8.3 Group 3: PH due to Lung Disease

Group 3 PH is primarily associated with chronic lung disease, most commonly COPD and ILD. Oxygen therapy is recommended as needed to promote normoxia. Additionally, pulmonary rehabilitation and physical therapy improve exercise tolerance and quality of life [14]. Results of PAH vasodilator agents in Group 3 PH have been mixed, with many studies finding neutral results and some showing potential for harm, such as a trial of riociguat in idiopathic interstitial pneumonia that found increased serious adverse events and mortality [46, 59]. After the INCREASE trial showed improved 6MWD compared to placebo inhaled treprostinil became the only PAH-specific medication approved for Group 3 PH due to ILD [60]. Treatment of Group 3 PH (outside of PH secondary to ILD) using vasodilator agents could worsen hypoxia and is not recommended [14].

Otherwise, treatments are targeted to the underlying etiology of lung disease. In select patient populations, surgical options such as bullectomy, lung volume reduction, and lung transplant may be considered [14].

Specific indications for lung transplant in Group 3 PH are the presence of moderate to severe PH in COPD and evidence of PH by echocardiography or RHC in ILD [2, 61].

### 8.4 Group 4: Chronic Thromboembolic Pulmonary Hypertension (CTEPH)

Chronic pulmonary emboli lead to the formation of organized thrombi and vascular remodeling which subsequently leads to increased pulmonary vascular resistance and Group 4 PH. Management includes screening appropriate patients with V/Q lung scanning, as patients with CTEPH are more likely to have an abnormal V/Q lung scan compared to an abnormal CT pulmonary angiography [6]. The mainstay of treatment is pulmonary endarterectomy, a surgical procedure that removes thrombi and thus relieves pulmonary artery obstruction and decreases pulmonary vascular resistance [2]. Balloon pulmonary angioplasty uses a catheter and balloon which gets inflated to dilate stenosed or thrombotic PA branches and augment blood flow. It is currently only recommended in patients with inoperable CTEPH or CTEPH that persists or recurs after endarterectomy [62].

Anticoagulation also plays an important role in preventing recurrent thromboembolism. Vitamin K antagonists (VKAs) such as Warfarin are well studied as are the gold standard but require frequent international normalized ratio (INR) monitoring and titration. Direct oral anticoagulants (DOACs) such as apixaban, rivaroxaban and dabigatran are being more widely used as they are easier to administer and do not require frequent lab monitoring. However, cost can be a prohibitive factor for some patients and there is limited evidence comparing them to VKAs [63].

When it comes to vasculature-modifying agents, prostacyclins have inconsistently demonstrated significant improvements in hemodynamic measurements and WHO functional class, indicating their potential use for CTEPH patients with inoperable or recurrent PH after invasive intervention. However, more research is needed to validate findings [64].

For patients who do not have anticipated improvement with the pulmonary endarterectomy, balloon pulmonary angioplasty is an option. It involves using a balloon to open residual stenotic and thrombosed pulmonary arteries. Medical therapy with riociguat, has been approved by the FDA for use in Group 4 PH to improve exercise tolerance and decrease pulmonary vascular resistance [65].

### 8.5 Group 5: PH Due to Miscellaneous/Unclear/Multifactorial Mechanisms

This group encompasses all other etiologies of pulmonary hypertension not otherwise mentioned above, including systemic, rheumatologic, hematologic and metabolic diseases. As such, treatment of PH in this group is highly specific to the underlying etiology [16]. As this group is heterogeneous at baseline, important notes of caution exist, for example, PDE5is increase the risk of pain crises in patients with sickle cell disease.

Regarding other etiologies of Group 5 PH, limited observational data suggest improvement in PH symptoms with tadalafil, macitentan, and selexipag due to T-cell large granular lymphocytic (LGL) leukemia. Though this therapy appeared useful in a case report, LGL leukemia is a relatively uncommon etiology for group 5 PH and data were very limited, further highlighting the need for a personalized approach to each patient [66]. Regarding more common etiologies of Group 5 PH, such as sarcoidosis, recent randomized clinical trial data have supported the use of ricioguat to slow progression of sarcoidosis-associated pulmonary hypertension [67]. However, this trial was small, consisting of only 16 patients, and riociguat has been suggested to increase harm in Group 3 PH, so its use here would be limited to those with Group 5 alone. As patients may fall into multiple groups of PH, use of riociguat would need to be carefully considered.

See Table 2 (Ref. [39, 40, 41, 42, 44, 48, 49, 50, 52, 60, 68, 69, 70, 71, 72, 73]) for a summary of important clinical trials and their findings. Importantly, psychosocial support should be considered alongside initiation of pharmacological therapies, as PH has significant effects on quality of life for both the patient and their family [2].

**Table 2.** **FDA-approved medications for PH**.

Drug (brand)	Pharmacologic class	FDA indication	Clinical trials
Ambrisentan (Letairis)	Oral endothelin-1 receptor antagonist	Group 1 to improve exercise ability and delay clinical worsening.	ARIES-1 and ARIES-2: randomized, double-blind, placebo-controlled study in 393 patients with PAH. Showed improvement in 6MWD and increased time to clinical worsening of PAH [41].
Bosentan (Tracleer)	Oral endothelin-1 receptor antagonist	Group 1 to improve exercise ability and decrease clinical worsening. In pediatric patients with PAH to improve exercise ability.	Study 351 and BREATHE-1: randomized, double-blind, placebo-controlled studies of 32 and 213 patients with WHO-FC III–IV PAH. Showed an increase in 6MWD and a reduction in rate of clinical worsening [39, 40].
Macitentan (Opsumit)	Oral endothelin-1 receptor antagonist	Group 1 to reduce risks of disease progression and hospitalization for PAH.	SERAPHIN: long-term, placebo-controlled study of 742 patients with WHO-FC II–IV PAH. Showed reduction in clinical worsening events [42].
Sildenafil (Revatio)	Oral PDE5 inhibitor	Group 1 to improve exercise ability and delay clinical worsening.	SUPER-1: randomized, double-blind trial of 277 patients with PAH WHO-FC II–III. Showed improvement in 6MWD [44].
Tadalafil (Adcirca)	Oral PDE5 inhibitor	Group 1 to improve exercise ability.	PHIRST-1: randomized, placebo-controlled study of 405 patients with PAH. Showed improved 6MWD and time to clinical worsening [68].
Riociguat (Adempas)	Oral soluble guanylate cyclase stimulator	Group 1 to improve exercise capacity, WHO-FC, and delay clinical worsening. Inoperable/persistent Group 4 to improve exercise capacity and WHO-FC.	PATENT-1: randomized, double-blind study of 443 patients with PAH. Showed improvement in 6MWD and WHO-FC [50].
			CHEST-1: randomized placebo-controlled study of 261 patients with CTEPH. Showed improvement in 6MWD, WHO-FC, and time to clinical worsening [69].
Epoprostenol (Flolan, Veletri)	IV prostacyclin analog	Group 1 to improve exercise capacity.	Barst *et al*. [70]: randomized, placebo-controlled study of 81 patients with PAH WHO-FC III or IV. Showed improvement in 6MWD and hemodynamics.
Iloprost (Ventavis)	Inhaled prostacyclin analog	Group 1 to improve exercise tolerance, symptoms, and lack of deterioration.	Olschewski *et al*. [71]: randomized, placebo-controlled study of 203 patients with PAH and CTEPH, WHO-FC III or IV. Showed improvement in exercise capacity and hemodynamics.
Treprostinil (Orenitram)	Oral prostacyclin analog	Group 1 to improve exercise capacity.	FREEDOM-M: randomized, placebo-controlled study of 228 patients with PAH. Showed improvement in 6MWD [72].
Selexipag (Uptravi)	Oral prostacyclin-receptor agonist	Group 1 to delay disease progression and reduce risk of hospitalization for PAH.	GRIPHON: double-blind, placebo-controlled, parallel group study of 1156 patients with PAH, WHO-FC I–IV. Showed reduction in disease progression events and hospitalization for PAH [48].
Treprostinil (Tyvaso, Yutrepia)	Inhaled prostacyclin analog	Group 1 to improve exercise ability, Group 3 to improve exercise ability.	TRIUMPH I: randomized, double-blind, placebo-controlled study of 234 patients with PAH, WHO-FC III. Showed an increase in 6MWD [49].
			INCREASE: randomized, double-blind, placebo-controlled study of 326 patients with PH-ILD. Showed an increase in 6MWD [60].
Macitentan-Tadalafil (Opsynvi)	Oral combination ERA and PDE5i	Group 1, chronic treatment in WHO-FC II–III.	A DUE: randomized, double-blind, adaptive, active-controlled, parallel-group study of 187 patients with WHO-FC II-III PAH. Showed greater reduction in PVR compared to single agent [73].
Sotatercept-csrk (Winrevair)	Activin signaling inhibitor	Group 1, to increase exercise capacity, improve WHO-FC, and reduce risk of clinical worsening events.	STELLAR (Phase 3)—demonstrated significant improvement in 6MWD and reductions in clinical worsening vs placebo [52].

PH, pulmonary hypertension; 6MWD, six-minute walk distance; PAH, pulmonary arterial hypertension; PDE5i, phosphodiesterase-5 inhibitor; ERA, endothelin receptor antagonist; WHO-FC, World Health Organization functional class; PH-ILD, pulmonary hypertension associated with interstitial lung disease; PVR, pulmonary vascular resistance; FDA, Food and Drug Administration.

## 9. Challenges

There are several challenges and gaps in clinical knowledge in the field of PH. As previously mentioned, significant delays in diagnosis are common and lead to poor prognosis and limitations for future treatment options, such as transplant considerations. A delay in diagnosis is more than one year on average and more than 3 years in 20% of patients, leading to incremental healthcare costs, productivity losses, and psychosocial burden [74]. Contributors include underconsideration of PH for common symptoms like dyspnea and fatigue and underutilization of key diagnostic testing such as TTE or RHC [75]. The American Society of Echocardiography emphasizes a stepwise, multimodal approach to improve diagnostic accuracy, acknowledging that referral to tertiary care centers or specialized PH centers is often necessary for complex cases and multidisciplinary care [2, 56]. These specialized centers allow for multidisciplinary care that may consist of cardiologists, pulmonologists, palliative care, physical therapy, and pharmacists. Expert PH centers are associated with improved outcomes including reductions in hospitalizations and mortality [76], however, the accredited centers in the United States are insufficient to meet the needs of this growing patient population and socioeconomic factors compound this disparity.

Some proposed interventions to overcome these gaps include dissemination of simplified PH resources to improve non-expert PH knowledge, establishing relationships between PH experts and community providers to bolster referral networks, using telehealth to mitigate geographic barriers, and building satellite sites to expand access to PH experts [77]. In 2024, members of the Lung Association and Pulmonary Hypertension Association compiled a 6-page “Guidance to the Guidelines” resource for the diagnosis and management of PH, which can be accessed online for free [78].

Disparities in PH exist regarding social determinants of health. There is underrepresentation of racial minorities in PH databases and clinical trials, limiting generalizability of findings and validity of guidelines based on evidence from these trials. In studies of PAH registries, Black patients are often younger, with higher comorbidity scores compared to white patients and are less likely to be prescribed index combination therapy for PAH [79, 80, 81]. In the US, minority populations are more likely to be under- or uninsured, limiting treatment options in PH, as many therapies cost hundreds to thousands of dollars per month without insurance, in addition to the cost of inpatient hospital stays and outpatient appointments. A study of PAH in the US found that out-of-pocket costs per patient per month ranged from $ 341 to $ 907 [82]. While a complex topic, these findings highlight a need for decreased healthcare costs and increased outreach to underrepresented populations for clinical trial enrollment.

Patients with PH must navigate life with an uncurable, progressive disease and the psychosocial burden cannot be overstated. Dyspnea, fatigue, and exercise intolerance limit patient independence and participation in society. Anxiety and depression are common [11], and the nature of PH as a chronic, progressive disease leads to poor quality of life. This is compounded by delays in diagnosis and complex treatment regimens commonly requiring close monitoring, long-term intravenous access, and significant caregiver support. Routine screening for psychosocial issues using assessment tools like the Cambridge Pulmonary Hypertension Outcome Review [2], support for mental health, and multidisciplinary care promote personalized medicine and improved long-term outcomes.

Major strides in PAH treatment development have occurred, with many clinical trials ongoing for PAH drugs targeting vascular remodeling, such as those for dichloroacetate, ranolazine, human histone deacetylase inhibitors, and trimetazidine [83, 84, 85, 86]. Despite advances in PAH treatments targeting endothelial dysfunction and vascular remodeling, limited treatment options remain for Group 2 through 5 PH. Group 2 PH is the most prevalent, yet most PAH-specific therapies are not recommended due to lack of efficacy and potential hemodynamic harm, and management focuses on optimizing the underlying cardiac pathology [2]. A phase 2 clinical trial is ongoing to study the use of a sotatercept analog in a subset of patients with left heart disease and combined pre- and post-capillary PH, but results have not yet been published [87]. Only riociguat has drug approval outside Group 1 [65]. Notably, the change in definition of PH occurred fairly recently and many clinical trials used the previous threshold of mPAP >25 mmHg when determining inclusion criteria [2]. Collectively, these gaps reflect the heterogeneous mechanisms, comorbid conditions, and limited large-scale evidence for targeted therapy. To address this, researchers have moved into exploring molecular targets and gene therapies as potential targets. Several promising strategies include targeting pulmonary artery smooth muscle cell hyperproliferation, the BMPR2 pathway which is involved in pulmonary vascular homeostasis, and vasoactive intestinal peptide which regulates vascular tone [88, 89].

Additional challenges facing the diagnosis and treatment of PH are several key differences between major society guidelines for diagnosis and management of PH, particularly between the ESC/ERS and the American Heart Association (AHA) guidelines. The ESC/ERS 2022 guidelines have more granular recommendations for risk stratification and prioritize referral to PH centers [2], while the AHA guidelines focus more on perioperative considerations [1]. More unified recommendations will lead to smoother diagnostic and referral pathways, improving access to care.

## 10. Future Directions 

In addition to the genetic and molecular targets above, future directions for PH research include high-quality investigations of artificial intelligence (AI) and wearable technologies for PH diagnosis and monitoring. AI-guided diagnosis in PH is emerging as a valuable adjunct for early detection, risk stratification, and phenotyping. TTE-based machine learning models can predict PH with high discrimination (area under the curve varied between 0.79–0.83) and may assist in classifying PH subtypes, expediting consideration of specific therapies [90, 91]. Deep-learning algorithms applied to chest radiographs have shown promise in detecting PH in a retrospective cohort study demonstrating a sensitivity of 0.902 and an AUC of 0.964 for PH detection [92]. One study funded by the company Eko used phonocardiograms generated from their digital stethoscope to estimate PA systolic pressure and used an AI algorithm to classify audio segments as either “normal” or “elevated” PA systolic pressure and thereby estimate mPAP and suggest for or against PH. The overall sensitivity and specificity were 0.71 and 0.73 for detecting a PASP >40 mmHg [93]. Using TTE values as validation introduces inherent uncertainty as TTE cannot definitively diagnose PH, so while this may be a useful adjunct in forming clinical suspicion for PH in resource-limited settings, it should not preclude further investigations [92]. Other algorithms are being developed using clinical and biomarker data for diagnostic assistance; Kovacs *et al*. [94] developed an algorithm utilizing electrocardiograms (EKGs), NT-proBNP, arterial O_2_ saturation, and WHO-FC to predict PH diagnosis, with a negative predictive value of 96% and positive predictive value of 92%. In clinical practice, using these non-invasive diagnostic modalities for diagnosis will require more standardized protocols, and improved internal and external validation.

Wearables and remote monitoring devices are rising in popularity, including commercial smartwatches or implantable devices such as CardioMEMS. These devices enable continuous measurement of physiologic parameters that can help contribute to PH diagnosis and monitor treatment response, however, current use is limited to adjunctive rather than definitive monitoring [95]. The CardioMEMS HF sensor, currently only approved in patients with NYHA class III HF, was utilized for monitoring patients with PAH and right-sided HF and indeed provided useful information to monitor response to PAH therapy, demonstrating potential utility in larger-scale clinical trials and potentially for prognostication and consideration of more aggressive or advanced therapies. Wearables enable continuous real-world data rather than episodic snapshots like serial RHC or TTE, guiding more responsive care. They also have the potential to notify providers of abnormal values or trends, prompting a communication with the patient. This would address several barriers to care including lack of transportation for appointments, living far from a PH center, and cost of serial imaging or testing.

## 11. Conclusion 

Major discoveries in recent years have deepened our understanding of the pathophysiology, diagnosis, and treatment of PH. With the newest changes in PH guidelines, many more patients can be classified as at risk of PH, and timely referral to specialist services can be initiated. Despite these progressions and hopeful future directions, there remain opportunities to streamline algorithms for diagnosis, monitoring, and intervention. An important underutilized resource is palliative care. Involving palliative care teams can alleviate challenging symptoms and provide emotional, spiritual, and psychosocial support for patients and their caregivers. Invasive palliative therapies such as atrial septostomy, whereby a right-to-left shunt is created to decompress the right heart, and right ventricular assist device placement, should also be considered for patients with advanced disease [96]. Additionally, the indications and timing of counseling regarding heart and lung transplantation warrant further study. To continue improving PH outcomes, emphasis should be placed on patient education, shared decision-making, and interdisciplinary care teams.
